# Heuristic Radio Access Network Subslicing with User Clustering and Bandwidth Subpartitioning

**DOI:** 10.3390/s23104613

**Published:** 2023-05-10

**Authors:** Marika Kulmar, Ivo Müürsepp, Muhammad Mahtab Alam

**Affiliations:** Thomas Johann Seebeck Department of Electronics, Tallinn University of Technology (TalTech), Ehitajate tee 5, 19086 Tallinn, Estonia; ivo.muursepp@taltech.ee (I.M.); muhammad.alam@taltech.ee (M.M.A.)

**Keywords:** 5G, RAN, slicing, UE clustering, performance evaluation

## Abstract

In 5G and beyond, the network slicing is a crucial feature that ensures the fulfillment of service requirements. Nevertheless, the impact of the number of slices and slice size on the radio access network (RAN) slice performance has not yet been studied. This research is needed to understand the effects of creating subslices on slice resources to serve slice users and how the performance of RAN slices is affected by the number and size of these subslices. A slice is divided into numbers of subslices of different sizes, and the slice performance is evaluated based on the slice bandwidth utilization and slice goodput. A proposed subslicing algorithm is compared with k-means UE clustering and equal UE grouping. The MATLAB simulation results show that subslicing can improve slice performance. If the slice contains all UEs with a good block error ratio (BLER), then a slice performance improvement of up to 37% can be achieved, and it comes more from the decrease in bandwidth utilization than the increase in goodput. If a slice contains UEs with a poor BLER, then the slice performance can be improved by up to 84%, and it comes only from the goodput increase. The most important criterion in subslicing is the minimum subslice size in terms of resource blocks (RB), which is 73 for a slice that contains all good-BLER UEs. If a slice contains UEs with poor BLER, then the subslice can be smaller.

## 1. Introduction

Network slicing in 5G cellular communication network is used to guarantee the service-level agreement (SLA) of a variety of user equipment (UE) instead of providing a best-effort networking service. Slices of network resources as logical separate networks can be created, modified and deleted automatically. The network configuration is dynamically adjusted to serve different groups of UEs. There are no upper bounds on the number of slices and slice size in the network.

Slice performance management is performed in the network slice lifecycle [1] by evaluating the slice performance. If a slice overload occurs, more resources are allocated to the slice. The computing, storage, and networking resources can be mapped to the QoS requirements by the orchestrator. The radio resources are limited and scarce. If additional resources are not available, then a slice overload can be avoided by not serving some UEs; however, then the slice SLA cannot be guaranteed. The mapping of radio resources to the QoS requirements is more complex. With subslicing the slice bandwidth is sub-partitioned into smaller parts to serve smaller UE groups.

From our previous work [2], it is known that the slice performance depends on how many subslices it is divided into. Given that different numbers of subslices affect the slice performance, a suitable number of subslices and subslice sizes exist. A subslicing algorithm can be created that uses found criteria on the subslice size and number of subslices that contribute to the slice performance improvement on a fixed slice bandwidth.

### 1.1. Related Work

The network slice subnet defined by 3GPP TS 28.530 [1] is a group of network functions that can be managed independently. It is easy to replace the words “network slice subnet instance” (NSSI) with a shorter word, “subslice”. The NSSI is a part of the core, RAN, edge, cloud, etc. network resources that can be separately incorporated into a slice to represent its part of network.

The slice identifier defined in 3GPP TS 23.501 [3] consists of a slice/service type (SST) that denotes standardized verticals or custom numbers and a slice descriptor (SD) that is optional and distinguishes multiple slices with the same SST. Reference [4] defined subslices as slices with the same SST but different SDs and evaluated the end-to-end performance with hundreds of slices in the system. The UE can connect to multiple slices, and slices can be created and deleted. However, RAN resources are assumed to be infinite, and the RAN is simulated using UERANSIM (where the physical layer is not implemented, and the radio interface is simulated over a UDP protocol), which means ideal radio quality and immediate transmission in the RAN is assumed.

Another study [5] proposed a subslicing method where the UE features were selected using a support vector machine (SVM) and the UEs were grouped based on the selected features using k-means. The number of subslices was determined based on the clustering quality measured with the Silhouette coefficient. However, this approach did not consider the performance when creating the subslices, which can lead to a poor performance for small subslices. Their simulations were conducted using Android UEs in Wi-Fi, and the performance of 5G-NR RAT was not evaluated.

On the other hand, Ref. [6] proposed a subslicing method where a subslice in a RAN is treated as a virtual cell that includes multiple physical cells. This approach aims to improve the slice performance by reducing the signaling required for cell handovers within the RAN subslice.

Lastly, a series of studies [7,8,9] attempted to address subslicing and its impact on performance. In [7], subslices were created for each vertical to include groups of UEs based on their similar SLA values. In [8], the focus was on optimizing the resource allocation for services, where UEs can connect to multiple subslices within a slice. The system load was defined as the range of the number of packets and packet sizes that will not fully utilize the allocated bandwidth. The related work on subslicing is compared in Table 1.

While all studies aimed to improve performance through subslicing, none of them considered the design of subslices that would achieve this goal in the RAN. It is important to note that the simulations used in these studies had a simplified RAN, and the requested rates were too variable to achieve a controlled load that would fully utilize the available bandwidth.

Key performance indicators (KPI) are used to evaluate the slice performance and trigger slice modification. The utilization of each resource as slice run-time KPIs are proposed in [10] to measure the slice performance. If the resource utilization exceeds a given high threshold (e.g., 80 %), then a slice overload can be detected, and slice modification can be triggered to add more resources or drop UEs. Similarly, in [11] high and low thresholds of computing resource utilization are used to trigger slice scaling, which is performed by adding or removing resources. Different values of slice overload thresholds are used. If new resources can be allocated, the slice overload threshold is 80–90%. If no resources are available and resources are taken from another less-loaded slice, the slice overload threshold is 60%. The slice overload threshold is useful for detecting slice overload earlier than it happens to avoid slice malfunction due to insufficient resources.

For subslicing, it is necessary to determine which UE should be served by which subslice. This can be achieved by clustering the UEs. UE clustering has been applied in several studies. In [12] the resource allocation scheme contains the allocation of a resource block (RB) on a time-frequency scale to a cluster of massive machine-type communications (mMTC) UEs in a MIoT slice; however, there are no details about how the UEs are clustered. As for the scheduler, one RB may be sufficient; however, one RB is too small for a subslice as a logical network on physical layer of 5G-NR. In [13] the UEs are clustered using the k-means clustering algorithm into inner-cell UEs and cell-edge UEs using the UE distance from the base station (BS). The bandwidth allocation is different for UE clusters: cell-edge UEs are allocated many smaller-bandwidth parts served by multiple close BSs, whereas inner-cell UEs are allocated bandwidth parts served only by the associated BS. In [14] the UEs are clustered by the UE position to decide how many small-cell BSs are needed to switch on or off to maximize the energy efficiency of the network. The clustering algorithm is based on fuzzy c-means clustering. Similarly, in [15] UEs are clustered by the UE position, and the optimal route for the flying BS is calculated for UE clusters. UE grouping or clustering has rarely been used for network slicing. In [16] a deep neural network is trained to classify UEs into enhanced mobile broadband (eMBB), MIoT, or ultra-reliable low-latency communication (URLLC) slices. In [17], the slice mobility is discussed. The UEs are grouped by the values of parameters that can indicate the UE behavior pattern in the network, that is, related to the association of the moving UE with different BSs. The operations with groups of UEs are related to slice life cycle management (LCM) and group handover, which is faster than triggering and performing the handover of each UE separately.

After initialization, the original k-means clustering algorithm [18] consists of three steps: distance calculation, cluster assignment, and a new centroid calculation. The algorithm finishes at convergence when the centroids do not move. The cluster size depends on the number of data points that are closer to the specific centroid and not to the other centroids. The cluster size is not limited. It can be empty or contain all data points. Furthermore, there are no outliers as each data point is assigned to a cluster.

K-means has many modifications, ranging from more unsupervised (no number of clusters given) to more constrained and balanced (all clusters of the same size). If an unsupervised k-means algorithm [19] is used, the algorithm determines the number of clusters to be created. This is based on data point similarity; however, we need to specify the desired number of subslices. The constrained k-means [20] algorithm enables the definition of constraints such as the minimum cluster size, and the optimization problem is solved using a linear programming method. Balanced k-means [21] results in clusters of equal size by defining artificial points close to the centroid and using the Hungarian algorithm to pair the data points with artificial centroid points. Agglomerative clustering algorithms begin with small clusters and attempt to merge similar clusters in each step. Finally, some clusters may include only one data point.

The subslicing has been implemented by grouping the services and UEs by their similarities. The effect on performance improvement has been noticed. The state of the art does not consider the following aspects:

- Regarding slice performance evaluation, most studies have considered throughput and/or delay as the main metrics. However, goodput (application-level throughput) should be taken into account, because throughput alone does not provide details about overheads (packet headers and packet retransmissions).
- Slice performance evaluation does not consider resource utilization, which is needed to understand the number and size of subslices to achieve the best performance.

### 1.2. Motivation and Problem Description

From our previous work, it can be seen that the slice performance depends on the number of subslices. The more subslices there are, the smaller one subslice is. How does the subslice performance depend on the subslice size?

When we see that subslices at some size have a better performance than others, then the slice could be subsliced into subslices of suitable size. What size and how many subslices could be created in the slice to improve the slice performance on a fixed slice bandwidth?

To evaluate how subslicing affects the slice performance, the following research problem is defined:

- **Input**: 275 UEs, 50 MHz (275 RBs), number of subslices in a slice, slice performance data (utilization, throughput, goodput, BLER) when the slice is not subsliced.
- **Decision**: Select the number of subslices to create and select the subslicing method.
- **Objective**: Improve the slice performance (reduce the bandwidth utilization and increase the slice goodput).

### 1.3. Contributions

The contribution is to answer the above-mentioned research questions and the specific outcome is as follows:

- Present the dependence of subslice performance on the subslice size.
- Propose a subslicing algorithm that prevents creating too-small subslices.
- Compare the slice performance, if it is subsliced using the proposed algorithm, equal UE grouping, or k-means UE clustering algorithms.

The remainder of this paper is organized as follows. In Section 2, the concept of subslicing is described and the performance of a subslice, depending on its size, is evaluated to determine the minimum subslice size. The proposed UE clustering algorithm is described in Section 3. Section 4 contains the slice simulations and slice performance-evaluation results when the slice is subsliced using three different subslicing algorithms. Finally, Section 5 concludes the paper.

## 2. Subslice Performance Evaluation at Different Subslice Sizes

In this section, the performance of the subslice at different sizes for the selected test case is evaluated.

### 2.1. Subslicing

Network slicing is a feature of 5G that is applicable to the 5G RAN with a stand-alone architecture. The RAN slice subnet, or the RAN slice, consists of a gNB, which is implemented as virtual network functions (VNFs) that use computing and storage resources to run and networking resources to enable connectivity between VNFs. The RAN slice also utilizes radio resources (frequency bandwidth) to transfer the UE data and control information using physical network functions, e.g., antennas [1].

To monitor the performance of the operational slices, a management closed control loop is utilized [22], which decides when the slice needs to be modified, and in case of slice overload, more resources can be added during the slice-modification phase [1]. This paper proposes subslicing as a means of slice modification to address a slice overload at a fixed slice bandwidth.

**Figure 1:**
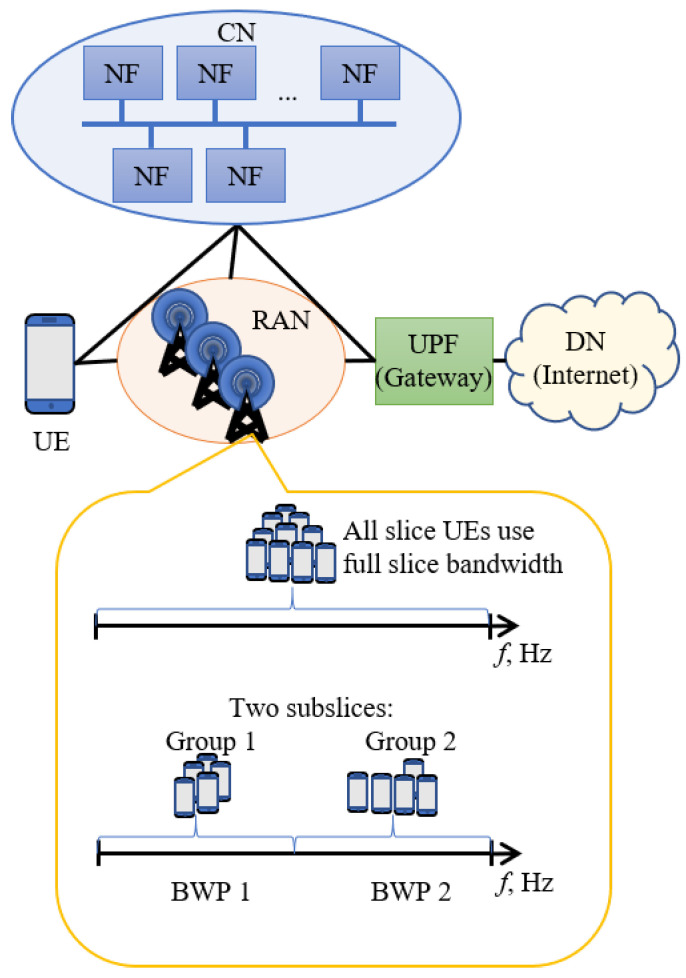
RAN architecture with slicing and subslicing.

Subslicing is a technique where the original slice bandwidth is divided into smaller parts. The slice UEs are grouped into smaller groups, and these smaller BWPs are allocated to these smaller UE groups. Figure 1 illustrates this approach. It is important to note that all subslices of a slice use slice VNFs and other slice resources.

To address a slice overload when there is insufficient bandwidth available, subslicing can be applied. The benefit of subslicing is that it can reduce the slice bandwidth utilization while simultaneously increasing the slice goodput, without requiring additional bandwidth allocation, thus ensuring rate requirements and serving more UEs.

**Figure 2:**
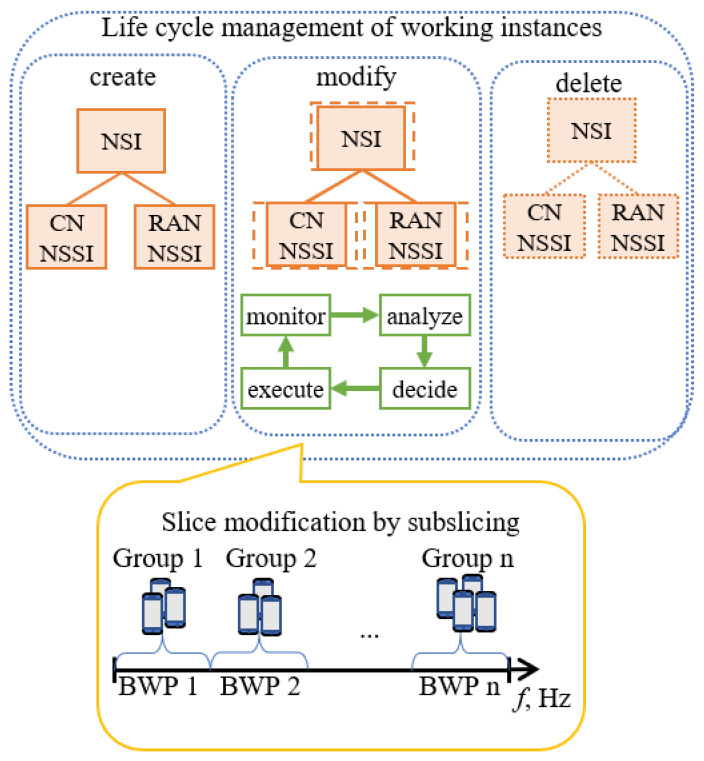
The subslice consists of a subset of slice UEs and a fraction of the slice bandwidth.

The life cycle of a working slice instance and the proposed RAN subslicing are illustrated in Figure 2. Similar subslicing has been carried out in [23], where dynamic inter-slice radio resource partitioning in the time-frequency plane is proposed. The optimization goal is to find the largest unallocated space. The bandwidth parts of the slices can be placed freely inside the time-frequency plane of the infrastructure radio resources.

The slice resource placement on the time-frequency plane is performed by inter-slice schedulers, but our subslicing framework resides on top of schedulers to subpartition the fixed bandwidth allocated to a slice to improve the slice performance.

### 2.2. Subslice Simulation Setup

The aim of this simulation is to study the dependence of subslice performance on the subslice size. The one-second working time of the subslice is simulated using the MATLAB 5G toolbox tool “NR Cell Performance Evaluation with Physical Layer Integration” [24]. The subslice has one RB of bandwidth resources allocated per UE in a subslice. In the simulations, the number of RBs and UEs in a subslice starts at four and increases by one until 275. All UEs are similar. The UE request rates are 500 kbps in the UL and 667 kbps in the DL. With these rates, it is possible to achieve approximately 80% utilization, and it allows seeing both an increase and decrease in utilization when subslicing. Each UE is positioned within 174 m (each of the three coordinates within 100 m) from the gNB and is expected to achieve a good BLER below 0.1. A value of 1500 bytes is the default value of the maximum supported packet size if the packet size is not specified in the slice template and 40 bytes is a short packet suitable for the MIoT slice [25]. The subslice simulation settings are presented in Table 2.

One parameter is the subband size, which depends on the allocated bandwidth part (BWP) in the RBs. The values of the subband size are defined in 3GPP TS 38.214 [26]. The subband size is used in the channel state information reporting. The other simulation parameters used in MATLAB are listed in Table 3. The subslice performance is measured by means of bandwidth utilization, subslice throughput (thr), and goodput (gdp) for UL and DL, and BLER for UL and DL.

### 2.3. Subslice Simulation Results

**Figure 3:**
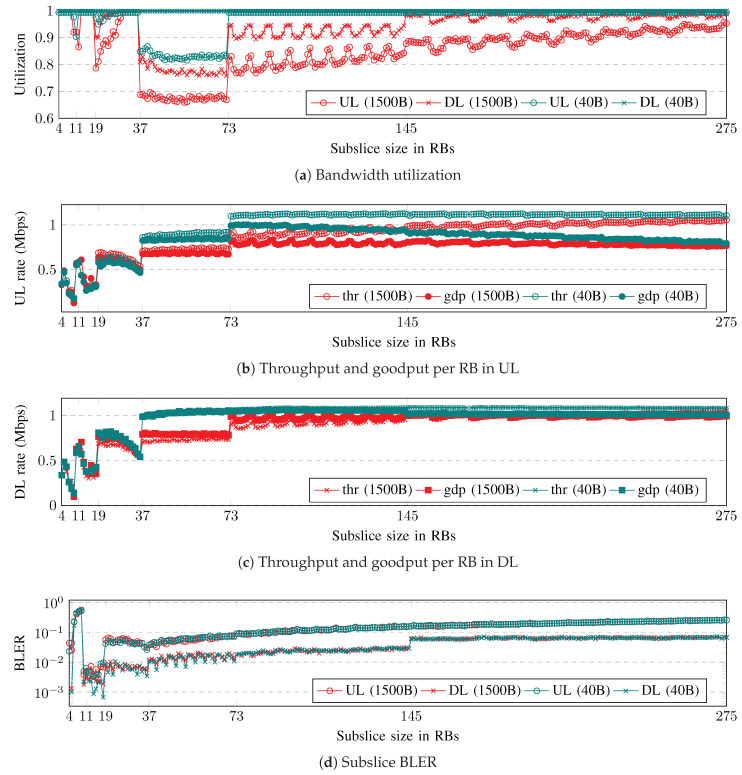
Subslice performance depending on subslice size in RBs. (**a**) Subslice bandwidth utilization, (**b**) UL and (**c**) DL throughput (thr) and goodput (gdp) per RB, (**d**) subslice BLER.

The subslice performance evaluation results, depending on the subslice size, are shown in Figure 3. The subslice bandwidth utilization, throughput, and goodput per RB and the average BLER are collected in both UL and DL. Regarding the packet size, longer packets result in lower bandwidth utilization, whereas shorter packets result in higher throughput and goodput. The BLER does not depend on the packet size, because the block size does not depend on the packet size.

**Figure 4:**
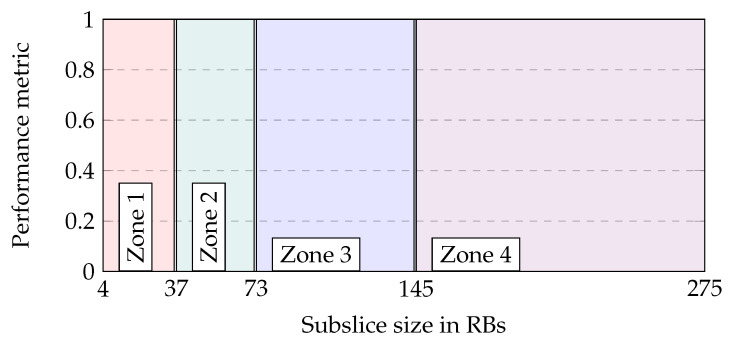
Performance zones depending on subslice size.

There are four different ranges of subslice sizes, called zones, in which the subslice performance is similar. The performance zones are shown in Figure 4 and the averages of the zone performance data are listed in Table 4.

The graph in Figure 3a displays the subslice performance in terms of the bandwidth utilization. The utilization is higher in DL than in UL, and with short packets, the utilization is higher than with long packets. For small subslices with sizes between 4 and 36 RBs (Zone 1), the bandwidth utilization is high. When the subslice size is between 37 and 72 RBs (Zone 2), the utilization drops to its lowest point. The utilization sharply increases to the slice overload threshold when the subslice size is between 73 and 144 RBs (Zone 3). Finally, when the subslice size is greater than 145 RBs (Zone 4), there is a significant boost in utilization.

Figure 3b,c illustrate the throughput (thr) and goodput (gdp) per RB for different packet sizes in UL and DL, respectively. The simulation results indicate that DL achieves a slightly higher throughput and goodput than UL. Additionally, the same requested data rate with short packets results in a higher goodput. The gap between the throughput and goodput contains the retransmission overhead, which is larger in UL than in DL. The small subslices in Zone 1 have a low throughput and goodput. As the subslice size increases to Zone 2, the throughput and goodput increase to a steady level, with short packets having higher throughput and goodput. In Zone 3, the throughput and goodput increase further, except for DL and short packets. In Zone 4 the goodput decreases in the UL.

The subslice BLER is shown in Figure 3d. Between subslice sizes of 19 and 37 RBs, the BLER gradually decreases before increasing slowly with increasing subslice size. The UL BLER is higher than that of the DL BLER. For the DL, the BLER is below 0.1 for subslice sizes in Zones 2–4. The UL BLER is also below 0.1 in Zones 2 and 3, but in Zone 4, the BLER increases gradually.

The zone boundaries refer to the BWP sizes where the value of a performance metric changes abruptly. This boundary coincides with the size of the RB group (RBG) changes, as specified in Configuration 1 in 3GPP TS 38.214 [26]. The Round Robin scheduler allocates RBGs for each UE for a slot time. The modulation and coding scheme (MCS) and code rate are selected based on the reported channel quality indicator (CQI). The transport block size (TBS) is calculated as specified in Sections 5.1.3.2 (DL) and 6.1.3.2 (UL) of 3GPP TS 38.214. The UEs in slices with smaller BWPs achieve a lower CQI. This is because a smaller BWP has fewer reference symbols available for channel estimation, which can lead to incorrect channel estimation and inappropriate TBS selection. If the channel is estimated to be better than its actual value, then a smaller TBS is selected, resulting in a lower achieved rate. If the channel is worse than the estimated value, a block error occurs. The performance pattern dependent on the BWP size can be repeated if the average MCS of the UEs is calculated for each BWP size, excluding the MCSs for the first three slots when the channel state information (CSI) is unavailable.

The Zone 1 subslice shows poor performance due to its high utilization and low goodput. Zone 2 has the lowest utilization and BLER. Zone 3 has a high goodput with short packets, and Zone 4 has a high goodput with long packets, but a high BLER in UL with both packet sizes. The selected minimum subslice size values are 37 and 73 RBs for the proposed subslicing algorithm, respectively. The former has better utilization, whereas the latter has better goodput.

Ref. [27] conducted a measurement campaign on a 5G-NR gNB using n78 (60 MHz TDD 4:1) with 4 × 4 MIMO for DL and 2 × 2 MIMO for UL. The results showed that a comparable result achieved without MIMO per RB is 0.8 Mbps for DL and 0.9 Mbps for UL. According to our simulation results, a slice with a size of 275 RBs (50 MHz) can achieve a throughput of around 1.1 Mbps per RB and a goodput of 0.8 Mbps per RB in UL and 1 Mbps per RB in DL.

## 3. Proposed User Clustering and Bandwidth-Allocation Algorithms for Enhanced Slice Performance

In this section, the proposed UE clustering with a bandwidth-allocation algorithm for subslicing is presented. The subslice performance results show that subslices that are too small will degrade the overall slice performance. The proposed clustering algorithm avoids creating clusters of UEs that are too small to allocate too few RBs for a subslice. In addition, a bandwidth-allocation algorithm for UE groups to allocate RBs proportional to the UEs in a group and a group BLER is proposed.

### 3.1. System Model

The slice is described as a bandwidth resource, a BWP in RBs, $N^{\left(RB\right)}$ and number of UEs, $N^{\left(UE\right)}$. The minimum subslice size requirement is denoted by $S_{min}$. The number of subslices requested is *K*.

The slice UEs are clustered for subslices. Each subslice *k* contains a subset (group) of slice UEs such that $N^{\left(UE\right)}=\sum_{k=1}^{K}N_{k}^{\left(UE\right)}$ and allocates a number of slice RBs such that $N^{\left(RB\right)}=\sum_{k=1}^{K}N_{k}^{\left(RB\right)}$ holds. All UEs are assumed to request the same rates. The given number of subslices and minimum subslice size constraint are assumed feasible. That is, with a given minimum subslice size constraint it is possible to create at least the requested number of minimum size or larger subslices, $K\leq \frac{N^{\left(RB\right)}}{S_{min}}$.

For clustering UEs into smaller groups for subslices the minimum cluster size can be calculated from the slice RBs and slice UEs:

(1) $$m_{min}=\left\lceil S_{min}\cdot \frac{N^{\left(UE\right)}}{N^{\left(RB\right)}}\right\rceil .$$

Then UEs are clustered into *K* clusters with minimum cluster size $m_{min}$.

The slice bandwidth $N^{\left(RB\right)}$ is allocated to UE clusters $N_{k}^{\left(UE\right)}$ in three steps: initially proportional to the number of UEs, secondly proportional to the number of UEs in a group and group BLER, and finally to subslices that are still too small.

The group BLER for UEs $u_{i}$ belonging to a cluster $C_{k}$ is calculated as the average of UE BLERs:

(2) $$BLER_{C_{k}}=\frac{\sum_{u_{i}\in C_{k}}BLER_{u_{i}}}{N_{k}^{\left(UE\right)}}.$$

### 3.2. Proposed UE Clustering Algorithm

**Figure 5:**
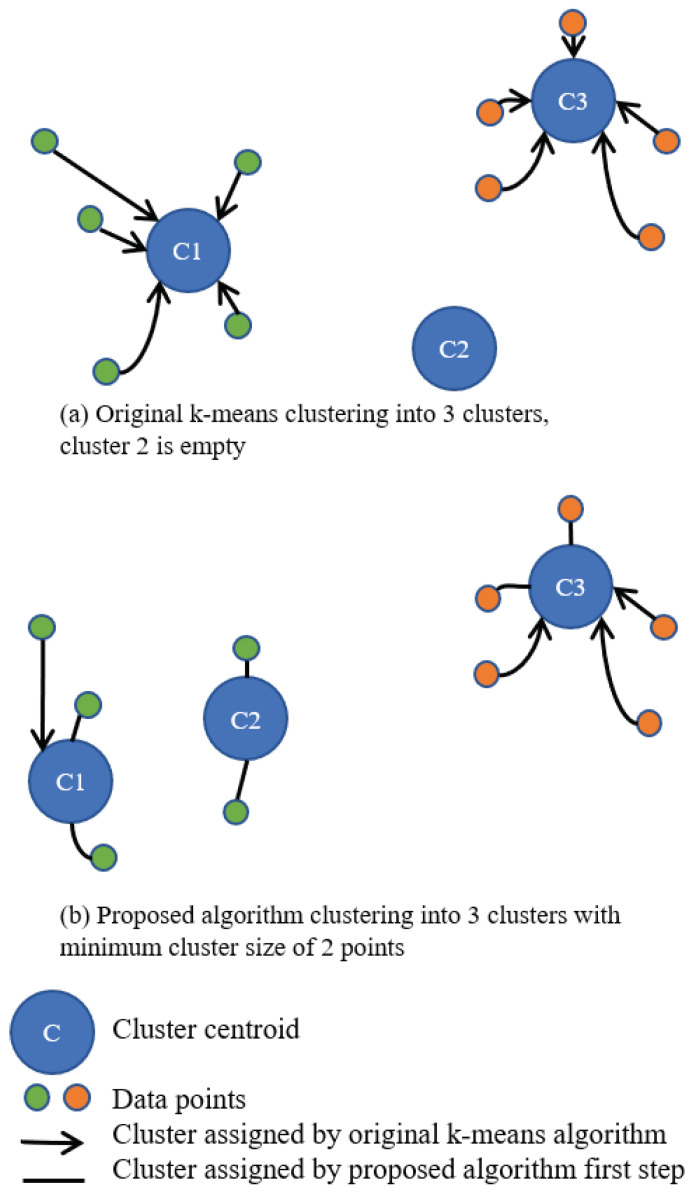
An example of how the proposed clustering algorithm clusters data points into three clusters with a minimum cluster size of 2. The data points cluster well into two clusters (green and orange).

The proposed algorithm is based on k-means clustering. The principle of the algorithm is illustrated using the example shown in Figure 5. In this example, the data points cluster well into two clusters, but it is necessary to cluster them into three clusters with a minimum cluster size of two. The proposed clustering algorithm is described next. In the first assignment step, each cluster takes the required number of data points closest to the cluster centroid. In the second assignment step, the unassigned data points are assigned to the cluster with the closest centroid (connections with arrows).

Let $u_{i}$ be the value of the metric used to cluster UEs, here UE BLER of the ith UE, i=1,...,N and $c_{k}$ be the centroid of cluster $C_{k}$, k=1,...,K. The distances are calculated using the Euclidean distance formula, (3),

(3) $$d_{k,i}=\sqrt{{\left(u_{i}-c_{k}\right)}^{2}}.$$

The distance matrix (4) is specified as follows:

(4) $$D=\left|\right|d_{k,i}{\left|\right|}_{n\times N^{\left(UE\right)}}.$$

where $d_{k,i}$ is the distance between UE *i* and the centroid of cluster *k*.

The original k-means algorithm [18] goes through the columns of the distance matrix and finds the minimum value of the column, and the row index designates the cluster to be assigned to the UE. This algorithm can leave some clusters empty in the worst-case scenario. To avoid empty clusters, the proposed algorithm goes through the rows and finds the minimum of each row for the column vector M and the index of UE, which has the minimum distance.

(5) $$M=\left|\right|m_{k}{\left|\right|}_{n\times 1}=\min_{i}\left|\right|d_{k,i}\left|\right|.$$

In each round, each cluster must select one UE from the column vector of minimum distances M. First, the UE with the minimum distance is selected by the cluster, and this cluster does not select another UE in this round.

(6) $$u_{i}\in C_{k},\;\mathrm{if}\;\min||m_{k}\left|\right|=d_{k,i}.$$

The value of the *k*th row in the column vector M is set to *∞* to prevent this value from being selected again as the minimum. The distance values of UE *i* in the distance matrix D are set to *∞* to prevent the UE from being assigned to another cluster.

The pseudocode for the modified k-means cluster assignment step is shown in Algorithm 1.
**Algorithm 1** Modified k-means cluster assignment step.1:**repeat**2:   **repeat**3:     Construct vector M from distance matrix D using Equation (5)4:     Find minimum in M and assign UE *i* to cluster *k*5:   **until** All clusters have a UE6:**until** All clusters have minimum number of UEs assigned

Next, when all clusters have collected the minimum number of UEs, the regular k-means cluster assignment is processed for the rest of the UEs still unassigned to a cluster:

(7) $$u_{i}\in C_{k},\;\mathrm{if}\;\min_{k}\left(|\right|d_{k,i}\left||\right)=d_{k,i}.$$

After cluster assignment, the new centroids are calculated as the mean of all points in the cluster. The convergence criterion is that the new centroid values remain the same as at the end of the previous iteration, as in the original k-means algorithm.

The complete proposed UE clustering algorithm is shown in Algorithm 2.
**Algorithm 2** Proposed algorithm for UE clustering.1:Initialization: Set *K* random centroids2:**repeat**3:   Calculate distance matrix D4:   **repeat**5:     Cluster assignment with modified k-means (Algorithm 1)6:   **until** All clusters have minimum number of UEs assigned7:   **if** unassigned UEs exist **then**8:     **repeat**9:        Cluster assignment with original k-means (Equation (7))10:     **until** All UEs have cluster assigned11:   **end if**12:   Calculate new centroids13:**until** convergence

### 3.3. Proposed Group Bandwidth-Allocation Algorithm

The slice bandwidth is allocated to the UE groups proportionally to the number of UEs in a group and the group BLER and taking into account that no subslice has a size smaller than the minimum subslice size criteria. Thus, the slice bandwidth part is allocated in three steps:

(8) $$N^{\left(RB\right)}=N_{1}^{\left(RB\right)}+N_{2}^{\left(RB\right)}+N_{3}^{\left(RB\right)}$$

and for subslices

(9) $$N_{k}^{\left(RB\right)}=N_{1,k}^{\left(RB\right)}+N_{2,k}^{\left(RB\right)}+N_{3,k}^{\left(RB\right)}.$$

Initially, all RBs are divided for the subslices and each subslice receives at least the initial number of RBs per UE. The initial allocation factor is calculated as follows:

(10) $$P_{1}=\frac{\left(N^{\left(RB\right)}/K\right)\cdot \left(N^{\left(RB\right)}/S_{min}\right)}{N^{\left(RB\right)}/N^{\left(UE\right)}},$$

which can be simplified to

(11) $$P_{1}=\frac{1}{N^{\left(UE\right)}}\cdot \frac{K\cdot S_{min}}{N^{\left(RB\right)}}$$

and the initial number of RBs of a subslice is

(12) $$N_{1,k}^{\left(RB\right)}=\left\lfloor N_{k}^{\left(UE\right)}\cdot P_{1}\right\rfloor .$$

The second allocation is proportional to the group BLER and the number of UEs in a cluster. The cluster with the better (smaller) BLER obtains fewer RBs per UE, and the cluster with the worse (larger) BLER obtains more RBs per UE in a cluster.

The BLER factor for a group is calculated from the group BLERs calculated using Equation (2) and the maximum BLER of group, which obtains a BLER factor of 1:

(13) $$f_{BLER_{k}}=\frac{BLER_{C_{k}}}{\max(BLER_{C_{k}})}.$$

The UE groups are not equal in size; therefore, the allocation factor $P_{2}$, which notes the RBs per UE proportional to BLER, is required. This is calculated from the group BLER factor and the number of UEs in a group.

(14) $$P_{2}=\frac{N^{\left(RB\right)}-N_{1}^{\left(RB\right)}}{\sum_{k=1}^{K}f_{BLER_{k}}\cdot N_{k}^{\left(UE\right)}}.$$

All required factors are calculated, and the remaining RBs from the first allocation are allocated to the groups using the following equation:

(15) $$N_{2,k}^{\left(RB\right)}=\left\lfloor N_{k}^{\left(UE\right)}\cdot f_{BLER_{C_{k}}}\cdot P_{2}\right\rfloor .$$

Finally, still too-small subslices will receive additional RB:

(16) $$N_{3,k}^{\left(RB\right)}=\left\{\begin{array}{cc} 1 & \mathrm{if}\;\left(N_{1,k}^{\left(RB\right)}+N_{2,k}^{\left(RB\right)}\right)<S_{min},\\ 0 & \mathrm{otherwise}. \end{array}\right.$$

The pseudocode of the bandwidth-allocation algorithm for the UE groups is shown in Algorithm 3:
**Algorithm 3** Proposed RB allocation for UE groups.1:Calculate initial allocation factor $P_{1}$ using Equation (11)2:Calculate RBs to subslices proportional to number of UEs in a cluster, $N_{1,k}^{\left(RB\right)}$ (Equation (12))3:**if**$N^{\left(RB\right)}-N_{1}^{\left(RB\right)}>0$ (unallocated RBs exist) **then**4:   Second allocation proportional to group BLER:5:   Calculate group BLERs (Equation (2))6:   Calculate group BLER factors (Equation (13))7:   Calculate allocation factor $P_{2}$ (Equation (14))8:   Calculate RBs to subslices proportional to group BLER and number of UEs in a cluster, $N_{2,k}^{\left(RB\right)}$ (Equation (15))9:**end if**10:**if** $N^{\left(RB\right)}-N_{1}^{\left(RB\right)}-N_{2}^{\left(RB\right)}>0$ (unallocated RBs exist) **then**11:   Add RB to too-small subslices, Equation (16)12:**end if**

### 3.4. Proposed Subslicing Algorithm

The full UE clustering with the bandwidth-allocation algorithm is as follows: first, the minimum cluster size for UE clustering is calculated using Equation (1). Then, UEs are clustered using Algorithm 2. Finally, the slice RBs are allocated to the UE groups using Algorithm 3.

## 4. Slice Performance Evaluation

We used the same simulation methodology as our previous work [2], described in Section 2.2, where the MATLAB tool is utilized to simulate individual subslices with specified parameters, including the number of RBs and UEs. In the first step, the slice UEs are all in one subslice, and all slice bandwidth is allocated to this one subslice. The slice performance and the block error ratio (BLER) for each UE are measured.

When the minimum subslice sizes are 73 and 37 RBs, three and seven subslices can be created, respectively. Otherwise, when there are more subslices, one or more subslices must be too small to degrade the slice performance. Smaller subslice sizes are tested to verify their effects on the poor slice performance.

### 4.1. Simulation Setup

Simulations are used to compare the slice performance if it is subsliced using equal UE grouping, k-means UE clustering, and the proposed subslicing algorithm. The equal-grouping algorithm groups the UEs into groups of as equal a size as possible and allocates RBs to the UE groups proportionally to the number of UEs in a group. This does not consider the diverse bandwidth requirements of UEs with different BLERs. K-means clusters the UEs into clusters of different sizes, and group-specific bandwidth allocation is not implemented. A subslice that is too small has a poor performance, which affects the slice performance, and the SLA of the UEs in a subslice that is too small will not be satisfied. The subslicing using k-means could be similar to [5], where the features are selected using SVM and the UEs are clustered using k-means. Although the number of features (clusters) is determined using SVM, the same weakness of k-means still remains: the cluster may be too small for achieving a good slice performance. The proposed algorithm uses modified k-means to create clusters that are not smaller than the minimum size requirement and allocates the bandwidth to the UE group proportionally to the number of UEs in a group and the group BLER. The subslices will not be too small, and the group BLER is considered in bandwidth allocation to the group. The slice does not require additional bandwidth to improve its performance.

The test cases are presented in Table 5. The slice is described as a bandwidth resource in RBs. The slice is allocated 275 RBs, as is the largest bandwidth 50 MHz or 100 MHz if using a subcarrier spacing of 15 or 30 kHz, respectively. If a larger bandwidth is needed, then carrier aggregation should be used. The slice resources are used by the UEs, and one UE is assumed to consume one RB, as in the previous simulations. The test cases include good-BLER, medium-BLER, and poor-BLER UEs. In order to obtain different BLER values with the channel model used, the UEs are located at different distances from the gNB.

From the previous section, it is clear that the subslice size should be at least 37 RBs to achieve a sufficient performance. For the simulations, the following numbers of subslices and minimum subslice sizes are selected for the proposed algorithm: three ($S_{min}=73$, the highest goodput expected), seven ($S_{min}=37$, the lowest utilization expected), 14 ($S_{min}=19$), 25 ($S_{min}=11$), and 68 ($S_{min}=4$). The last three subslice sizes are simulated to verify their effects on the poor slice performance. K-means is not used if 68 subslices need to be created. MATLAB cannot simulate BWP<4 RBs. Dividing 275 RBs into 68 BWPs, the subslices are at least four RBs, and only three subslices can be five RBs. It is difficult to achieve many small clusters of almost equal size by clustering.

The subslicing framework works on top of the scheduler, and the direct signal-quality parameters may not be available. The parameter to cluster UEs was selected to use BLER because it characterizes the signal quality for the UE better than the UE distance from the BS. Similar UEs based on their signal quality in the same subslice could have fairness in transmission. Any other parameter that characterizes the need for additional bandwidth for packet retransmission can be used for UE clustering.

Other simulation parameters are listed in Table 3. Performance data is collected for each subslice and combined to obtain the slice performance data. Each simulation is performed ten times. The mean values and 95% confidence intervals are calculated using MATLAB and its functions mean and fitdist, respectively.

### 4.2. Results

The slice performance is measured with the slice bandwidth utilization and achieved slice throughput and goodput. In addition, the average slice BLER is collected. Finally, the slice performance improvement achieved by subslicing compared to not subslicing is discussed.

The horizontal axis of the graphs show how many subslices the slice has been subsliced into. The vertical axis shows the performance metric.

#### 4.2.1. Slice Bandwidth Utilization

**Figure 6:**
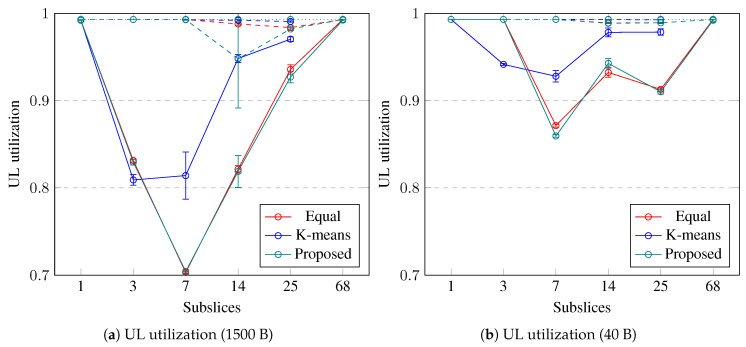
Simulation results on utilization in UL. Error bars show a confidence interval of 95%. Solid lines show slices containing good-BLER UEs, dashed lines show slices containing medium-BLER UEs, dotted lines show slices containing poor-BLER UEs.

The slice bandwidth utilization in the UL is shown in Figure 6. If the slice is not subsliced, the UL bandwidth utilization is 100%. The utilization decreases when the slice is subsliced into a few subslices, but if there are more than seven subslices, then the utilization in UL increases back to 100%. The slice bandwidth utilization in the UL decreases to 70% if 1500-byte packets are used, whereas 40-byte packet utilization decreases to 85%. Equal UE grouping and the proposed subslicing algorithm decrease the slice utilization more than k-means UE clustering. If the UE BLER is worse (dashed and dotted lines), subslicing does not decrease the slice bandwidth utilization. To improve the slice bandwidth utilization in UL by up to 41% by subslicing, the slice should contain good-BLER UEs that use longer packets. Equal UE grouping and the proposed algorithm can be used to perform subslicing into no more than seven subslices with a minimum subslice size of 37 RBs.

**Figure 7:**
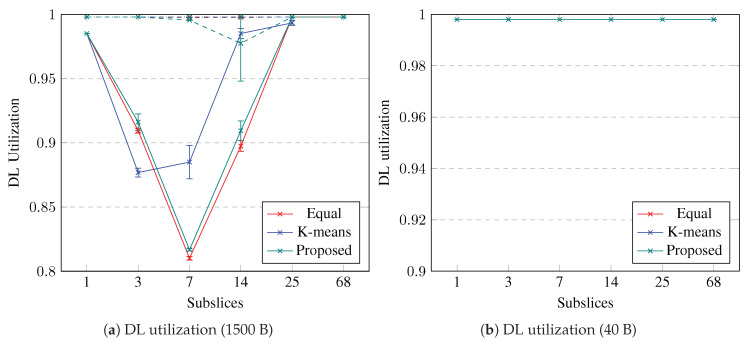
Simulation results on utilization in DL. Error bars show a confidence interval of 95%. Solid lines show slices containing good-BLER UEs, dashed lines show slices containing medium-BLER UEs, dotted lines show slices containing poor-BLER UEs.

The slice bandwidth utilization in DL is shown in Figure 7. If the slice is not subsliced, the DL bandwidth utilization is close to 100%. The utilization decreases when the slice is subsliced into a few subslices, but if there are more than seven subslices, then the utilization in UL increases back to 100%. The slice bandwidth utilization in DL decreases to 80% if 1500-byte packets are used, whereas for 40-byte packets, the utilization does not decrease at all. Equal UE grouping and the proposed subslicing algorithm decrease the slice utilization more than k-means UE clustering. Similar to UL, if the UE BLER is not good, then subslicing does not decrease the slice bandwidth utilization in DL. To improve the slice bandwidth utilization in DL by up to 22% by subslicing, the slice should contain good-BLER UEs that use longer packets. Equal UE grouping and the proposed algorithm can be used to perform subslicing into no more than seven subslices with a minimum subslice size of 37 RBs.

#### 4.2.2. Slice Throughput and Goodput

**Figure 8:**
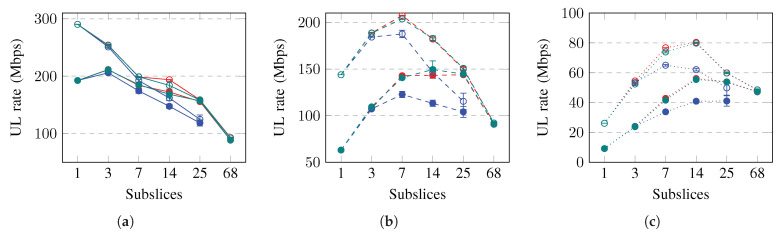

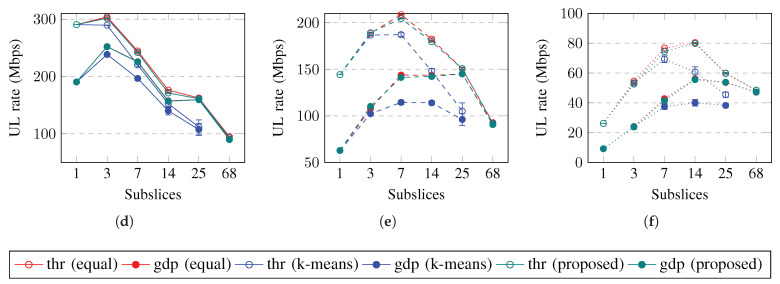
Simulation results on throughput (thr) and goodput (gdp) in uplink (UL). Error bars show a confidence interval of 95%. Solid lines show slices containing good-BLER UEs, dashed lines show slices containing medium-BLER UEs, dotted lines show slices containing poor-BLER UEs. (**a**) UL capacities (1500 B, good-BLER). (**b**) UL capacities (1500 B, medium-BLER). (**c**) UL capacities (1500 B, poor-BLER). (**d**) UL capacities (40 B, good-BLER). (**e**) UL capacities (40 B, medium-BLER). (**f**) UL capacities (40 B, poor-BLER).

The achieved throughput and goodput for a slice in the UL are shown in Figure 8. The slice throughput and goodput in the UL initially increase and then decrease when the slice divided into more subslices. The rates achieve maximum values when the slice is subsliced into three, seven, or 14 subslices if the slice contains good-BLER, medium-BLER, or poor-BLER UEs, respectively. Subslicing increases the goodput even more if the packet size is short, for example, 40 B. The achieved rates are higher when subslicing is performed using equal grouping or the proposed algorithm. Subslicing increased the achieved UL rates by up to 9%, 58%, and 84% if the UE BLER is good, medium, or poor, respectively. To increase the slice goodput in the UL by subslicing, the equal UE grouping or proposed algorithm should be used to subslice the slice into three, seven, or 14 subslices. Subslicing improves the slice goodput more if the UEs have worse BLER and use short packets.

**Figure 9:**
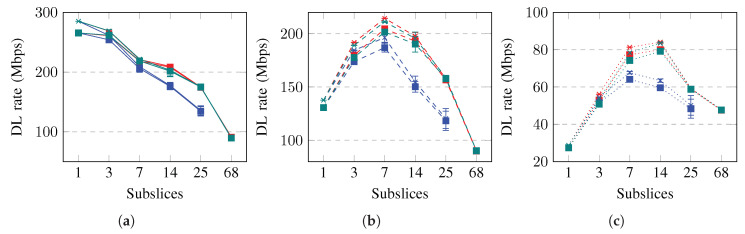

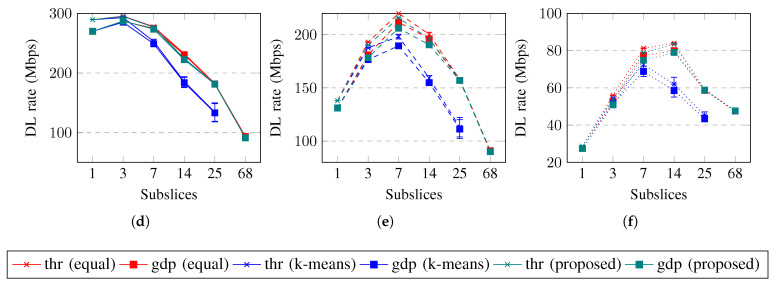
Simulation results on throughput (thr) and goodput (gdp) in downlink (DL). Error bars show a confidence interval of 95%. Solid lines show slices containing good-BLER UEs, dashed lines show slices containing medium-BLER UEs, dotted lines show slices containing poor-BLER UEs. (**a**) DL capacities (1500 B, good-BLER). (**b**) DL capacities (1500 B, medium-BLER). (**c**) DL capacities (1500 B, poor-BLER). (**d**) DL capacities (40 B, good-BLER). (**e**) DL capacities (40 B, medium-BLER). (**f**) DL capacities (40 B, poor-BLER).

The achieved throughput and goodput for a slice in DL are shown in Figure 9. The slice throughput and goodput in the DL change less than those in the UL. Similarly, in DL, the rates achieve maximum values when the slice is subsliced into three, seven, or 14 subslices if the slice contains good-BLER, medium-BLER, or poor-BLER UEs, respectively. Subslicing increases the DL goodput if the packet size is short and when subslicing is performed using equal grouping or the proposed algorithm. Subslicing increases the achieved DL rates by up to 6%, 38%, and 66% if the UE BLER is good, medium, and poor, respectively. To increase the slice goodput in DL by subslicing, equal UE grouping or the proposed algorithm should be used to subslice the slice into three, seven, or 14 subslices. Subslicing does not improve the slice goodput in DL if the UEs use long packets and have good BLER.

#### 4.2.3. Slice BLER

**Figure 10:**
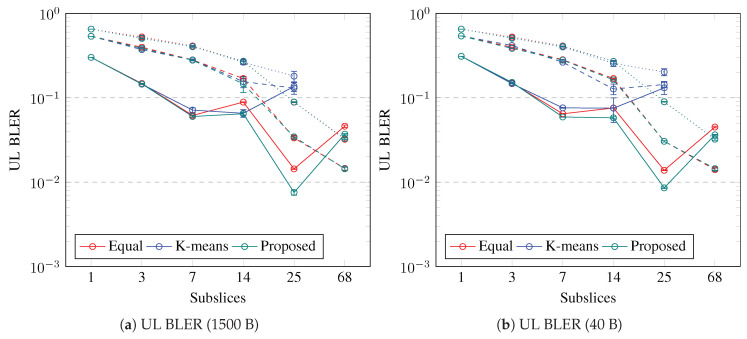
Simulation results on UL BLER. Error bars show a confidence interval of 95%. Solid lines show slices containing good-BLER UEs, dashed lines show slices containing medium-BLER UEs, dotted lines show slices containing poor-BLER UEs.

**Figure 11:**
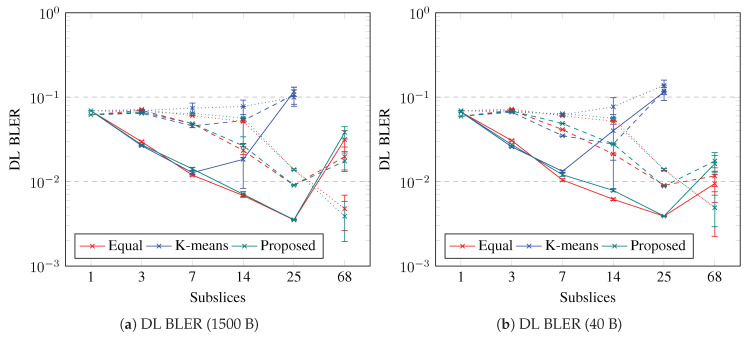
Simulation results on DL BLER. Error bars show a confidence interval of 95%. Solid lines show slices containing good-BLER UEs, dashed lines show slices containing medium-BLER UEs, dotted lines show slices containing poor-BLER UEs.

The slice BLER is the average of the UE BLERs. The slice BLER is shown in Figure 10 and Figure 11 for the UL and DL, respectively. The slice BLER in both the UL and DL is decreasing if the slice is subsliced into up to seven subslices. The slice BLER is similar for both packet sizes, except for DL with k-means UE clustering, in which short packets and 14 subslices of DL BLER are the lowest. The slice BLER in UL is a minimum if the slice is subsliced into two, seven, or 25 using the proposed algorithm; however, if 14 subslices are used, k-means achieves the best UL BLER. The slice BLER in DL is a minimum if the slice is subsliced into 25 using the proposed algorithm or equal UE grouping; however, if k-means UE clustering is used, then seven or 14 subslices achieves the best DL BLER with 1500-B and 40-B packet sizes, respectively. Subslicing enables a decrease in the BLER, especially for slices with good-BLER UEs; however, to decrease the slice BLER, if the slice contains medium- or poor-BLER UEs, more subslices are necessary. The slice BLER in the UL decreases with subslicing. If seven or fewer subslices are needed for good-BLER UEs, then using the proposed algorithm, the BLER in the UL improves the most. The slice BLER in DL can be decreased by subslicing into at least seven subslices if the slice contains good-BLER UEs. If equal UE grouping and the proposed algorithm are used for subslicing a slice that contains good-BLER UEs, the number of subslices can be up to 25. Subslicing can improve the slice BLER if equal UE grouping or the proposed algorithm is used to create 25 or 68 subslices for slices that contain medium-BLER or poor-BLER UEs, respectively. The slice BLER in UL is above 0.1 and it decreases below this value if the slice contains all good-BLER UEs and is subsliced into seven subslices. If the slice contains UEs with worse BLER, then BLER<0.1 is when 25 subslices are created using equal grouping or the proposed algorithm. In DL, the BLER is mostly below 0.1, except when 25 subslices are created using k-means UE clustering.

#### 4.2.4. Slice Performance Improvement

The slice performance is improved by subslicing if the slice bandwidth utilization decreases and achieved slice goodput increases. The percentage decrease in utilization is added to the percentage increase in goodput and compared to a slice not subsliced; that is, the number of subslices is one.

**Figure 12:**
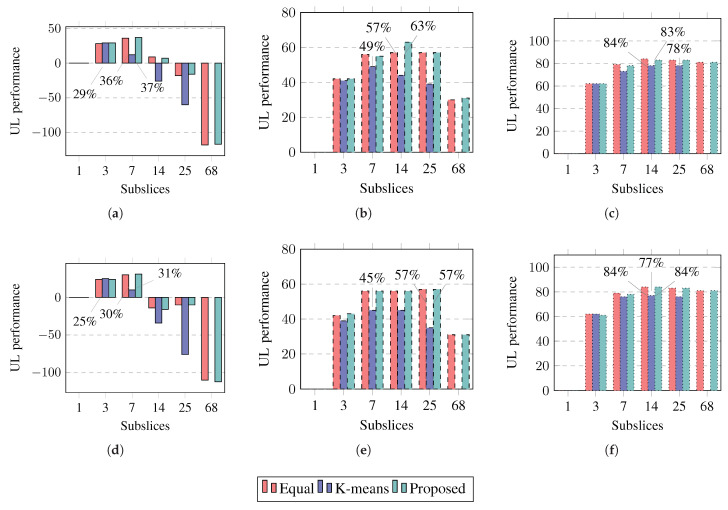
Simulation results on performance improvement percentages in UL compared to slice not subsliced. Solid lines show slices containing good-BLER UEs, dashed lines show slices containing medium-BLER UEs, dotted lines show slices containing poor-BLER UEs. (**a**) UL performance (1500 B, good-BLER). (**b**) UL performance (1500 B, medium-BLER). (**c**) UL performance (1500 B, poor-BLER). (**d**) UL performance (40 B, good-BLER). (**e**) UL performance (40 B, medium-BLER). (**f**) UL performance (40 B, poor-BLER).

The slice performance improvement in the UL is shown in Figure 12. The results show that the slice performance in UL can be improved another 6% more if a longer packet size is used. Equal UE grouping and the proposed algorithm improve the slice performance more than k-means UE clustering. Subslicing improves the slice performance in UL by up to 37%, 63%, or 84% if the slice contains good-BLER, medium-BLER, or poor-BLER UEs, respectively. To improve the slice performance in the UL by subslicing, equal UE grouping or the proposed algorithm should be used to subslice the slice into seven or 14 subslices. If UEs have worse BLER, more subslices can be recommended.

**Figure 13:**
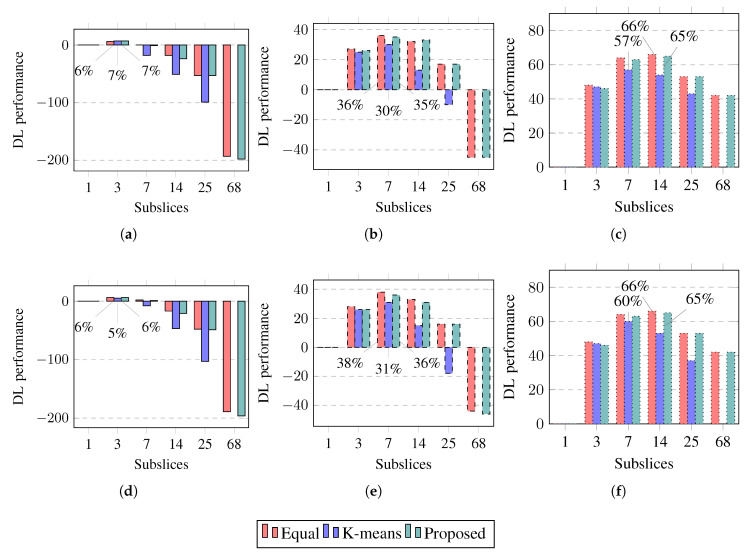
Simulation results on performance improvement percentages in DL compared to slice not subsliced. Solid lines show slices containing good-BLER UEs, dashed lines show slices containing medium-BLER UEs, dotted lines show slices containing poor-BLER UEs. (**a**) DL performance (1500 B, good-BLER). (**b**) DL performance (1500 B, medium-BLER). (**c**) DL performance (1500 B, poor-BLER). (**d**) DL performance (40 B, good-BLER). (**e**) DL performance (40 B, medium-BLER). (**f**) DL performance (40 B, poor-BLER).

The improvement in the slice performance in DL is shown in Figure 13. The slice performance in DL can be improved to a lesser extent than that in UL. The slice performance improvement in DL is similar for both packet sizes and is similar to UL and equal UE grouping, and the proposed algorithm improves the slice performance more than k-means UE clustering. Subslicing improves the slice performance in DL by up to 7%, 38%, or 66% if the slice contains good-BLER, medium-BLER, or poor-BLER UEs, respectively. To improve the slice performance in DL by subslicing, equal UE grouping or the proposed algorithm should be used to subslice the slice into three, seven, or 14 subslices if the slice contains good-BLER, medium-BLER, or poor-BLER UEs, respectively.

#### 4.2.5. Algorithm Performance

The performance of the proposed subslicing algorithm was compared with that of equal UE grouping and k-means UE clustering. The evaluation of the subslicing algorithms was based on the measurement of the slice performance improvement achieved and the computational time required for the algorithm to calculate the subslice settings.

The performance improvement percentages for UL and DL, averaged across all UE types, are presented in Table 6. The results indicate that creating seven subslices using equal grouping or the proposed algorithm achieves the greatest improvement in slice performance. On average, subslicing improved the slice performance by over 40% compared to a non-subsliced slice.

**Figure 14:**
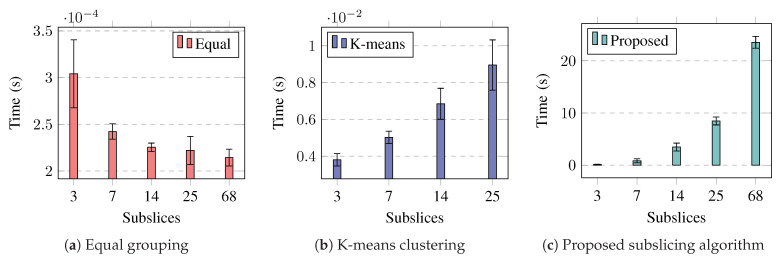
Measured time it takes to create sublices—group/cluster slice UEs and subpartition slice bandwidth. Error bars show a confidence interval of 95% from 60 runs.

To evaluate the complexity of different subslicing algorithms, we measured the time it took to run the MATLAB implementation. Each algorithm ran 60 times, with ten runs per test case. Figure 14 shows the measured average time, and the error bars indicate a 95% confidence interval. When using equal grouping, the time required to calculate the subslice configuration did not depend on the number of subslices. However, other algorithms took more time as the number of subslices increased. Although the proposed algorithm required the most time, the subslices it created outperformed those created using k-means UE clustering.

In conclusion, subslicing can be recommended to improve the slice performance. Subslicing is more effective in reducing the slice bandwidth utilization with long packets and increasing the slice goodput with short packets. The UL benefits more from subslicing due to a greater reduction in the UL bandwidth utilization. Both random UE grouping and the proposed algorithm are suitable for subslicing, but k-means UE clustering creates a set of clusters where some clusters are too small, which results in a poor subslice performance and degrades the slice performance. Subslicing is more effective in improving the slice performance in the UL and for UEs whose BLER is not good. The recommended number of subslices to be created is higher if the slice contains UEs whose BLER is worse.

Dividing a network slice into suitably sized subslices can have positive system implications, as it can improve the slice performance and provide service categories with specific resource allocations to satisfy particular requirements. However, subslicing also has negative implications. For example, it can increase the network management complexity and require additional resources to calculate the subslice configurations.

## 5. Conclusions

In this paper, subslicing was investigated as a method to improve the RAN slice performance. The present literature does not evaluate the number and size of subslices required to achieve a slice performance improvement in 5G-NR. The slice bandwidth was subpartitioned and allocated to smaller groups of slice UEs. The subslices were simulated individually, and the performance data were combined to represent the slice performance.

Our work demonstrates the positive effect of subslicing on slice performance. Moreover, our work has determined the criteria to achieve a slice performance improvement. The benefit of subslicing is the efficient use of radio resources; however, it requires computing and storage resources to create subslices.

The simulations were performed with all UEs having similar rate requirements and BLER. This enables the evaluation of how the performance of slice UEs can be improved by subslicing. However, the subslicing algorithm can be improved for a realistic case in which the UEs have different BLERs and requested rates. Then, the subslices for the UEs with worse BLERs can be smaller.

The results show that the slice performance depends on the number of subslices and the subslice performance depends on its size. The minimum subslice size requirement ranged from 11 to 73 RBs. The lower the UE BLER, the higher the minimum subslice size. The number of subslices with which the slice performance can be improved is higher if the BLER of the UEs is higher.

Future work will add the decision of subslicing to the toolbox for slice modification to improve the slice performance without additional bandwidth.

## Figures and Tables

**Table 1:** Comparison of related work in subslicing.

Paper	How Subslicing Is Performed	Benefits	Limitations
[7]	Three verticals each contain subgroups of services. UEs grouped by similar SLA values	Performance improvement achieved by subslicing: increased SNR and throughput.	The planned use of capacity varies between underload and half of the full load. The effect of the number of subslices on slice performance has not been evaluated.
[8]	A subslice is a logical group of services that is associated with a single UE. The UE can connect to multiple subslices.	The performance improvement achieved by subslicing: increased throughput and energy efficiency.	The planned use of capacity is variable between underload and close to full load.
[9]	One subslice can serve not only UEs with similar SLAs, but also a mixed set of UEs with different throughputs.	The performance improvement achieved by subslicing: increased throughput.	The planned use of capacity cannot be evaluated using the given data.
[5]	UEs are clustered by the similarity of their requirements.	Subslicing results in decreased bandwidth consumption, improved load balancing, improved latency and heterogeneity, and improved energy efficiency.	Planned underload, RAN simulated as Android UEs in Wi-Fi. The proposed subslicing method does not avoid creating subslices that are too small.
[6]	In the RAN subslice, the virtual cell covers multiple physical cells, and the UE by mobility allocates the virtual cell.	The latency and throughput can be improved because UE handover is performed faster.	Evaluation of just signalling for handover. The UE can select subslices without constraints on the subslice performance.
[4]	The slice with identifier SST contains all slices with the same SST and different SDs as subslices.	Performance improvement was achieved by subslicing.	The RAN was simulated as ideal.
This paper	The slice bandwidth is subpartitioned, slice UEs are grouped, and bandwidth subpartitions are allocated to UE groups. **The number and sizes of subslices are determined with the aim of achieving better performance than without subslicing.**	Slice performance can be improved by reducing bandwidth utilization and increasing goodput if the subslices are not too small. Simulations were performed using **5G-NR and close to the full capacity** of the allocated bandwidth. UE requested rates are sufficient to utilize one RB per UE.	Simulations were performed under the assumptions that all UEs are similar to their requirements and capabilities. The proposed algorithm requires significant computational resources.

**Table 2:** Subslice data.

Parameter	Value	
Number of UEs in a subslice	{4–275}	
Number of RBs allocated to subslice	{4–275}	
Subslice modification for next step	Increase UEs by one and increase RBs by one	
Subcarrier spacing	15 kHz	
UE rate requirements	UL 500 kbps, DL 667 kbps	
**Subband size**		
**Size of BWP in RBs**	**Subband size specified in TS 38.214**	**Subband size used in simulations**
4–23	-	4
24–72	4, 8	8
73–144	8, 16	8
145–275	16, 32	16

**Table 3:** MATLAB Toolbox settings.

Parameter	Value
Carrier frequency	3 GHz
Channel model (for both UL and DL)	CDL-C
PUSCH preparation time for UEs	200 μs
Logical channels per UE	1
RLC entity type	UM bidirectional
Duplex mode	FDD
Scheduler strategy	Round Robin
Length of scheduling cycle	1 frame
RB allocation limit UL	same as RBs for subslice
RB allocation limit DL	same as RBs for subslice
Simulation time	1 s
Subslice simulation tool from MATLAB 5G Toolbox	NR Cell Performance Evaluation with Physical Layer Integration [24] R2021b

**Table 4:** Average subslice simulation results for subslice size ranges. Best values of each parameter are shown in bold.

Zone	Subslice Size (RBs)	Average Utilization				Average Throughput				Average Goodput				Average BLER			
		1500 B		40 B		1500 B		40 B		1500 B		40 B		1500 B		40 B	
		UL	DL	UL	DL	UL	DL	UL	DL	UL	DL	UL	DL	UL	DL	UL	DL
1	4–36	0.949	0.988	0.986	0.998	0.522	0.571	0.485	0.586	0.493	0.566	0.457	0.581	0.081	0.056	0.079	0.053
2	37–72	**0.675**	**0.777**	**0.831**	0.998	0.726	0.804	0.896	1.044	0.68	0.791	0.838	1.031	**0.057**	**0.015**	**0.061**	**0.013**
3	73–144	0.815	0.923	0.993	0.998	0.915	1.000	**1.115**	1.082	**0.796**	0.975	**0.97**	**1.057**	0.122	0.024	0.122	0.024
4	145–275	0.905	0.978	0.993	0.998	**1.014**	**1.065**	1.113	**1.087**	0.786	**0.994**	0.863	1.015	0.205	0.064	0.206	0.064

**Table 5:** Simulation settings.

Parameter	Value
Slice bandwidth	275 RBs (49.5 MHz)
Subcarrier spacing	15 kHz
Number of UEs is the slice	275
Number of subslices and minimum subslice sizes	3, if $S_{min}=73$ 7, if $S_{min}=37$ 14, if $S_{min}=19$ 25, if $S_{min}=11$ 68, if $S_{min}=4$
Parameter to cluster UEs	BLER
UE distance from gNB (m): all good-BLER UEs, all medium-BLER UEs, all poor-BLER UEs	. 1–275 1001–1275 6001–6275
UE rate requirements	UL 500 kbps, DL 667 kbps
UE packet sizes	{1500 B, 40 B}

**Table 6:** The average slice performance improvement summed for UL and DL and compared with that when the slice was not subsliced.

Algorithm	3 Subslices	7 Subslices	14 Subslices	25 Subslices	68 subslices
**Equal**	35.3	45	36.5	24.2	−32.7
**K-means**	34.6	34.8	18.4	−4.9	N/A
**Proposed**	34.8	44.3	35.5	24.3	−33.8

## Data Availability

The data presented in this study are available on request from the corresponding author.
